# Fiber orientation in a whole mouse heart reconstructed by laboratory phase-contrast micro-CT

**DOI:** 10.1117/1.JMI.7.2.023501

**Published:** 2020-03-04

**Authors:** Marius Reichardt, Mareike Töpperwien, Amara Khan, Frauke Alves, Tim Salditt

**Affiliations:** a University of Göttingen, Institute for X-Ray Physics, Göttingen, Germany; b Max Planck Institute for Experimental Medicine, Göttingen, Germany; c University of Göttingen, Department of Hematology and Oncology, Göttingen, Germany

**Keywords:** laboratory propagation-based phase contrast, heart tissue, cardiomyocytes, orientation, anisotropy, degree of filament alignment

## Abstract

**Purpose:** We present a phase-contrast x-ray tomography study of wild type C57BL/6 mouse hearts as a nondestructive approach to the microanatomy on the scale of the entire excised organ. Based on the partial coherence at a home-built phase-contrast μ-CT setup installed at a liquid metal jet source, we exploit phase retrieval and hence achieve superior image quality for heart tissue, almost comparable to previous synchrotron data on the whole organ scale.

**Approach:** In our work, different embedding methods and heavy metal-based stains have been explored. From the tomographic reconstructions, quantitative structural parameters describing the three-dimensional (3-D) architecture have been derived by two different fiber tracking algorithms. The first algorithm is based on the local gradient of the reconstructed electron density. By performing a principal component analysis on the local structure-tensor of small subvolumes, the dominant direction inside the volume can be determined. In addition to this approach, which is already well established for heart tissue, we have implemented and tested an algorithm that is based on a local 3-D Fourier transform.

**Results:** We showed that the choice of sample preparation influences the 3-D structure of the tissue, not only in terms of contrast but also with respect to the structural preservation. A heart prepared with the evaporation-of-solvent method was used to compare both algorithms. The results of structural orientation were very similar for both approaches. In addition to the determination of the fiber orientation, the degree of filament alignment and local thickness of single muscle fiber bundles were obtained using the Fourier-based approach.

**Conclusions:** Phase-contrast x-ray tomography allows for investigating the structure of heart tissue with an isotropic resolution below 10  μm. The fact that this is possible with compact laboratory instrumentation opens up new opportunities for screening samples and optimizing sample preparation, also prior to synchrotron beamtimes. Further, results from the structural analysis can help in understanding cardiovascular diseases or can be used to improve computational models of the heart.

## 1 Introduction

Heart contractility, as one of the most important physiological functions, relies on an intricate, hierarchical molecular and cellular architecture. The macroscopic structure of the heart and the inner structure of the heart muscle has been known for more than a hundred years from classical anatomy and histology. However, the invasive nature of histological sectioning impedes the reconstruction of the detailed three-dimensional (3-D) arrangement of cardiomyocytes and myofibrils for the whole organ. Accordingly, the 3-D structure of the heart muscle is not completely understood, and there have been many different attempts to describe it, such as for example the controversial helical ventricular myocardial band^1^ or the idea of a complex 3-D mesh of cardiomyocytes surrounded by a fibrous matrix.^2^^,^^3^ Verification of these fundamental models requires high-resolution imaging of the heart structure. Ultrasound and diffusion tensor magnetic resonance imaging (MRI) are incapable of resolving the cellular structures of the entire heart with a resolution in the range a few micrometers.^4^ Conventional absorption-based x-ray imaging results in low contrast since the x-ray absorption of heart tissue is relatively small compared with mineralized tissues, such as bone. One way to increase contrast is the use of metal-based stains, such as iodine.^5^^–^^7^ To increase contrast for unstained tissue, its phase-shifting properties can be exploited for contrast formation in weakly absorbing objects. For soft tissue, the real-valued decrement δ of the refractive index n=1−δ+iβ is by 1 to 3 orders of magnitude larger (depending on tissue composition and x-ray wavelength λ) than the imaginary part β. Phase-contrast x-ray imaging therefore offers superior image quality and in particular visibility of small features down to the cellular and subcellular scale, while keeping the penetration depth at the scale of the whole organ. The overall potential of the method has already been convincingly lined out using synchrotron radiation (SR) with a parallel beam geometry.^8^^–^^13^ In prior work, we could show that even subcellular features in heart tissue biopsies can be resolved^14^ by cone beam phase-contrast tomography with SR.

In this work, we explore an approach to small animal heart imaging based on compact laboratory instrumentation. Firstly, this offers broader accessibility of the method, including further clinical settings in the future. Secondly, high end SR beamtimes can be better prepared if different samples can be screened and sample preparation protocols can be optimized beforehand using laboratory instrumentation. In particular, we show that the image quality and resolution are high enough to deduce the 3-D (pseudo-) vector field of heart tissue. It is worth noting that the structure of the heart poses a multiscale challenge for structure analysis, based on the different hierarchies: heart, cardiac mesh, aggregation of myocyte chains (aggregated units), connective tissue, myocyte chain, single cardiomyocyte, myofibril, and finally the sarcomere as the molecular machine underlying contractility. Here, we present phase-contrast reconstructions of whole mouse hearts with effective pixel sizes around 5  μm. Based on the reconstructed electron density, the orientation of the muscle fiber bundles can be determined. To this end, we compare two different subvolume-based algorithms. The first algorithm is based on the Fourier transform.^15^^,^^16^ Its implementation and validation was one of the main goals of this work. By analyzing the Fourier space of small subvolumes, the fiber orientation, the degree of filament alignment, and structural information as local thickness of single muscle fiber bundles can be obtained. Results from this algorithm are then compared with a more established approach based on the local gradient of the reconstructed electron density, which was already applied to high-resolution synchrotron data.^8^^,^^9^^,^^17^

The rest of the paper is organized as follows. Following the introduction, the procedure of sample preparation, data acquisition, phase reconstruction, and tomographic reconstruction is described in Sec. 2. The quality of the 3-D electron density obtained for the different preparations is then evaluated in terms of resolution, signal-to-noise ratio (SNR), and preservation of the cardiac structure in Sec. 2.3. In Sec. 3, the Fourier-transform-based algorithm, including the analysis of orientation, degree of alignment, and fiber thickness, is introduced and compared with the gradient-based approach. In Sec. 3.4, the work is summarized and further possibilities for data analysis and possible applications as well as restrictions of this procedure are pointed out.

## 2 Three-Dimensional Structure of a Whole Mouse Heart

### 2.1 Sample Preparation

**Fig. 1:**
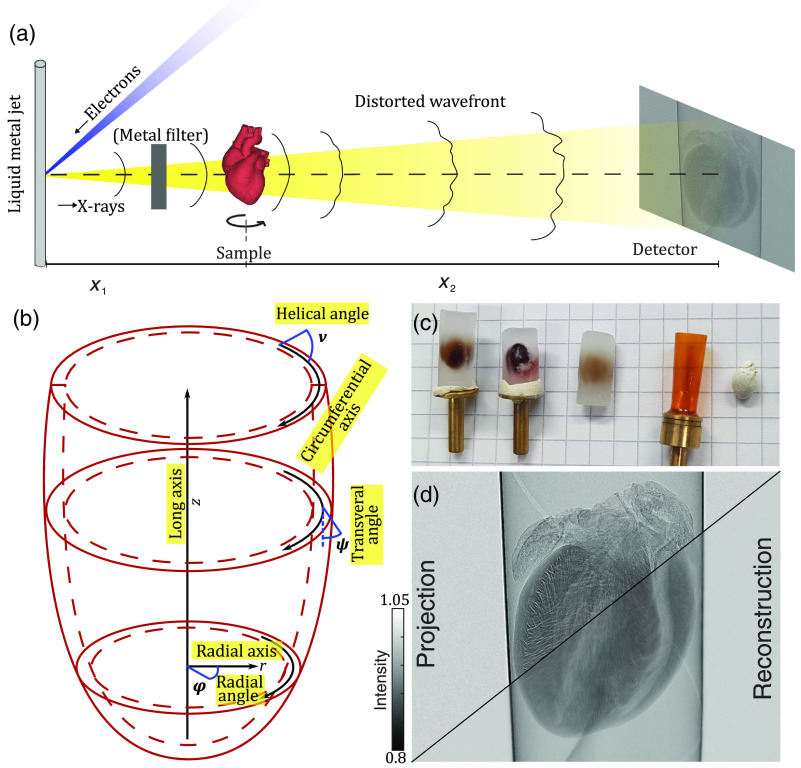
(a) Sketch of the laboratory LMJ μ-CT setup with electron beam sketched in blue and x-ray beam in yellow. The emission spectrum can be filtered (i.e., prehardened) by metal foils. The sample is placed at a distance $x_{1}$ behind the source spot and projections are recorded with a flat panel detector at distance $x_{2}$. (b) Definition of the coordinate system. Point in the heart is parameterized by cylindrical coordinates, i.e., z along the long axis positioned in the center of the left ventricle, azimuthal angle φ, and radial distance r. The fiber orientation is parameterized by the in-plane angle ψ and the helical angle ν. (c) Image of the different samples. From left to right: unstained, iodine-, and PTA-stained paraffin-embedded hearts, polyimide tube for sample mounting, and EOS heart. (d) Empty beam-corrected projection acquired at the laboratory setup and the corresponding phase reconstruction.

Figure 1 presents the imaging setup, coordinate system, and representative images of the samples. The heart structure of wild type C57BL/6 mice was investigated, using five different preparations, in view of comparing contrast and SNR:

1. paraffin embedding,^18^
2. paraffin embedding with an iodine stain,^6^
3. paraffin embedding with a phosphotungstic acid (PTA) stain,^18^^,^^19^
4. embedding in liquid ethanol,^20^ and
5. evaporation-of-solvent (EOS) method.^21^

The whole explanted hearts were washed in phosphate-buffered saline and fixated in 4% formaldehyde solution for 24 h. The stains were administered in solution by diffusion of the staining agent, with incubation times of 24 h (iodine) or 7 days (PTA). Afterward, the samples were dehydrated in an increasing series of ethanol. For paraffin embedding, the hearts were transferred to xylene and cast in paraffin. Excess paraffin was removed with a scalpel before imaging the sample. The heart embedded in 100% ethanol solution was mounted in a polyimide tube (Kapton, Professional Plastics, Inc.) with an inner diameter of 6 mm and a wall thickness of 75 μm. The EOS method was performed following the protocol described in Ref. 21. It is based on dehydration of the fixed sample in ethanol and removal of lipids using xylene similar to the classical histology procedure. The major difference is the evaporation of xylene instead of the transfer to liquid paraffin. The evaporation of xylene results in tissue shrinkage but does not alter the gross morphology.^21^ The sample was also mounted in a polyimide tube with an inner diameter of 6 mm. An image of the different samples is shown in Fig. 1(c).

### 2.2 Data Acquisition and Phase Reconstruction

Data acquisition was performed at a laboratory liquid metal jet (LMJ) μ-CT (Excillum Metaljet-D2) as shown in Fig. 1(a). The x-rays were produced by electrons focused on a liquid galinstan (68.5% Ga, 21.5% In, and 10% Sn) jet with a diameter of ∼180  μm. For the experiments in this work, the electrons were focused down to $10\times 40\;{\mu m}^{2}$ resulting in an effective spot size of $∼10\times 10\;{\mu m}^{2}$. The source was operated with a power of 100 W at an acceleration voltage of 70 keV. For samples with a higher absorption, the emission spectrum of galinstan with a characteristic energy at 9.25 keV ($\mathrm{Ga}\;K_{\alpha }$) was prehardened by a metal filter (35  μm Ag, 25  μm Ni) resulting in a peak energy of the $K_{\alpha }$ emission line at 24.2 keV.^22^^–^^24^ The metal filter was installed behind the exit window of the source. The samples were mounted on a motorized triaxial translation and rotation stage (SmarAct GmbH, PI miCos GmbH) at a distance $x_{1}=10$ to 13 cm behind the source. Two additional translation stages (PI miCos GmbH) below the rotation axis allow the positioning of the rotation axis along the optical axis. For image acquisition, a flat panel detector with a GsOS:Tb-scintillator screen (Dexela CMOS, PerkinElmer Inc.) consisting of 1536×1944  pixels with an isotropic pixel size of dx=75  μm on a motorized three directional translation system was placed at a distance of $x_{2}=150$ to 180 cm behind the sample. The cone beam geometry of the setup allows for customizing zoom and field of view by geometric magnification $M=(x_{1}+x_{2})/x_{1}$ and leads to effective pixel sizes of $dx_{\mathrm{eff}}=(dx/M)=5.2$ to 5.5  μm for the different data sets.^25^ Due to a small effective source spot, the effects of source blurring are relatively small. Considering the cone beam geometry, projections were taken from 1001 equidistant angular positions over 185 deg for all data sets. To increase the signal without saturating the detector, five acquisitions at each position were recorded and averaged. For the metal-stained samples, the increased photon absorption of the tissue leads to a decrease in detectable photons at the detector plane. Most notably, low-energy photons are absorbed by the sample. To reduce beam-hardening artifacts, the spectrum of the emitted x-rays was prehardened by a metal filter and the acquisition time was adapted to keep the photon flux of the empty beam comparable between the measurements. The acquisition and geometric parameters are given in Table 1 and were chosen in view of magnification/sample size and visibility of phase-contrast effects (edge enhancement).

**Table 1:** Acquisition parameters for the tomographic measurements at the laboratory μ-CT setup.

Preparation method	Paraffin	Paraffin + PTA	Paraffin + iodine	Ethanol	EOS
$x_{1}$ (cm)	10.00	10.00	12.75	12.75	12.75
$x_{1}+x_{2}$ (cm)	144.60	144.60	174.40	174.40	180.50
Effective pixel size (μm)	5.2	5.2	5.5	5.5	5.3
Beam prehardening	No	Yes	Yes	No	No
Acquisition time (s)	5×0.6	5×1.6	5×1.6	5×0.6	5×0.6

There are different approaches for the phase retrieval of the single projections, such as Paganin’s single material object approach,^26^^,^^27^ the contrast transfer function,^28^ or iterative algorithms.^29^ In this work, the Bronnikov-aided correction (BAC) was used.^30^ The BAC approach provides optimal results for the imaging of weak objects, such as soft tissue in the direct contrast regime, in particular at low coherence sources.^31^ In addition to the phase-shifting properties of an object, the BAC also takes the absorption into account. This leads to a reconstruction of the image properties based on coupled (mixed) phase and absorption contrast, without losing resolution due to a blurring of the reconstruction. Figure 1(d) shows an empty beam-corrected projection from the analysis of the EOS preparation on the left side. The corresponding reconstruction in which the edge enhancement was corrected is given on the right side of the image. For small propagation distances x between the object and the detector, the transport of intensity equation (TIE) can be linearized,^32^ and the intensity can be written as $I_{x}=I_{0}[1-(x2\pi /\lambda )\nabla_{⊥}^{2}\phi ]$. In a first step, the phase ϕ of a projection is approximated by the modified Bronnikov algorithm,^33^ based on the TIE inversion of a pure phase object

$$\phi =\frac{2\pi }{\lambda x}\cdot F^{-1}[\frac{F\left[I_{x}-1\right]}{{\left|k\right|}^{2}+\alpha }].$$ (1)

To account for singularities at k=0, an absorption-dependent regularization parameter α is introduced to prevent strong artifacts and blurring. These would amplify low spatial frequencies in the image and would inevitably corrupt these Fourier components resulting in strong artifacts and blurring. The assumption of a weak object leads to blurred reconstructions of objects with non-negligible absorption. To resharpen the image, a second reconstruction step is applied, in which a weakly absorbing and homogeneous object is assumed and the (effective) intensity distribution at the object plane is reconstructed from the approximated phase ϕ via

$$I_{x_{1}}=\frac{I(\Delta x)}{\left(1-\gamma \nabla_{⊥}^{2}\phi \right)},$$ (2)

where γ is a second α-dependent regularization parameter.^31^ All parameters for the phase reconstruction of the different samples are shown in Table 2.

**Table 2:** List of the phase retrieval parameters for the BAC.

Embedding	Staining	α	γ
Paraffin	None	0.02	0.0690
Paraffin	PTA	0.05	0.0690
Paraffin	Iodine	0.03	0.0785
Ethanol	None	0.02	0.0785
Air (EOS)	None	0.02	0.0850

### 2.3 Evaluation of the Tomographic Reconstructions

**Fig. 2:**
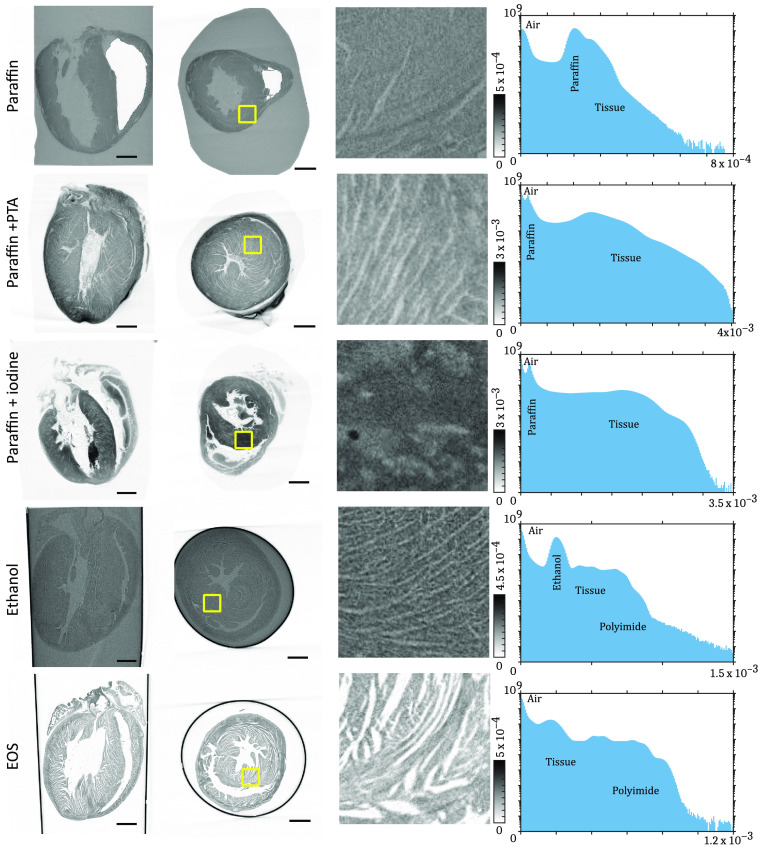
Results of the tomographic reconstructions of the different sample preparations. For each row, the following items are shown from the left to right: a slice of the reconstructed volume along the long axis of the heart, a slice along the short axis, a zoom of the marked area of the interventricular septum, and a histogram of all gray values within the volume. Scale bars: 1 mm.

The tomographic reconstruction was performed using the ASTRA toolbox.^34^ Additionally, a ring correction^35^ was performed. To characterize the 3-D structure of the heart, it is necessary to image the whole organ with sufficient resolution and contrast to identify the direction of the muscle fibers. Therefore, the results are evaluated with respect to resolution, SNR, and preservation of the tissue structure. Orthogonal slices of all tomographic reconstructions of the hearts are shown in Fig. 2. Each row of the figure shows a slice of the 3-D volume along the long and short axes of the heart. In the lateral slices, an area of the interventricular septum is marked in yellow and a zoom of the region is shown on the right. Furthermore, a histogram of all gray values within the 3-D volume is given on the right. The values are defined by the tomographic reconstruction of the effective absorption coefficient μ

$$\ln[\frac{I(r)}{I_{0}}]=-\mu Ndx_{\mathrm{eff}},$$ (3)

with the normalized intensity $I(r)/I_{0}$ of the projections and N=1944 is the number of horizontal detector pixels. The resolution of the respective data sets was calculated by the Fourier shell correlation (FSC)^36^ of two independent reconstructions obtained by splitting the projections in two sets with odd and even angles. The SNR was determined by

$$\mathrm{SNR}=\frac{{\overset{¯}{A}}_{\text{signal}}-{\overset{¯}{A}}_{\text{noise}}}{\sigma_{\text{noise}}}$$ (4)

where ${\overset{¯}{A}}_{\text{signal}}$ is the mean amplitude of 100 voxels from a selected area of tissue of the centered slice with the highest signal, ${\overset{¯}{A}}_{\text{noise}}$ is the mean amplitude of the surrounding medium, and $\sigma_{\text{noise}}$ is its standard derivation. The resolution obtained by FSC and the SNR for each preparation is given in Table 3.

**Table 3:** List of the SNR as well as the resolution obtained at the laboratory μ-CT for different sample preparations.

Embedding	Staining	SNR	Resolution (μm)	Normalized resolution ($dx_{\mathrm{eff}}$)
Paraffin	None	8.8	8.15	1.57
Paraffin	PTA	25.3	6.80	1.31
Paraffin	Iodine	29.2	9.10	1.65
Ethanol	None	5.1	8.78	1.60
Air (EOS)	None	13.0	6.20	1.17

The overall structure of the hearts can be seen in all reconstructions, but the visibility of detailed structures within the cardiac tissue differs based on the different preparations. The resolution of all data sets is in the range below 2 pixel and mostly depends on the geometric magnification of the setup. Differences of the normalized resolution can be explained by the contrast and quality of phase reconstruction. Thus, the main focus is set on the evaluation of contrast and the conservation of the cardiac structure. In terms of contrast, metal-based staining of tissue samples result in the highest SNR. Due to the penetration with high Z-elements, the electron density of the tissue increases and thus the interactions with the x-ray in terms of absorption and phase-shifting properties increases. Consequently, the difference between tissue and background leads to a better contrast. The SNR of both metal-stained tissues is over 25 and about three times higher compared with other preparation, but there are differences in the details of the tissue structure in both stains, as can be seen in the zoom of the radial slices in Fig. 2. PTA can bind with a higher affinity to various cardiac tissue structures, such as collagen, fibrin, and other fibers of connective tissue.^37^ On the contrary, iodine seems to lack this specificity and, hence, results in blurry reconstructions and randomly clusters at a few regions of the tissue. Furthermore, salt crystals from the buffer can be identified within the left ventricle. The data set of the dehydrated heart in ethanol has the lowest SNR; nevertheless single muscle fibers can be identified. The noise could be suppressed by a longer acquisition time and the contrast enhanced by a longer incubation in ethanol.^20^ To keep the results comparable, experiments with higher acquisition times or incubation in ethanol are not shown in this study. For the preparation of the samples in liquid, it is mandatory to prevent bubbles because even small bubbles in the liquid can grow and cause motion artifacts. For the preparations of the hearts mounted in paraffin, air bubbles in the heart chamber do not cause motion artifacts due to the stability of the paraffin. Nevertheless, especially for the unstained tissue, the sharp edges from air to paraffin lead to strong scattering of the x-ray and result in small artifacts in phase reconstruction. The air bubbles can be removed by vacuumizing the tissue in liquid paraffin. The EOS preparation leads to a very structured reconstruction of the heart tissue. The removal of water and lipids lowers the absorption of the sample but enables an enhanced contrast between tissue and air. The single muscle bundles separate from each other due to the dehydration and shrinking of the sample. This not only leads to an increase in contrast but also allows for analyzing the thickness of these bundles. Since the cardiomyocytes primarily separate from each other and do not rupture or break, the principal structure of the heart should not be affected by this preparation. Further, the EOS reconstruction has a high SNR and the structure of the tissue is not affected by any staining agent. Hence, the set is used for further analysis.

## 3 Orientation of the Cardiomyocytes in the Heart

### 3.1 Fourier-Transform-Based Algorithm

**Fig. 3:**
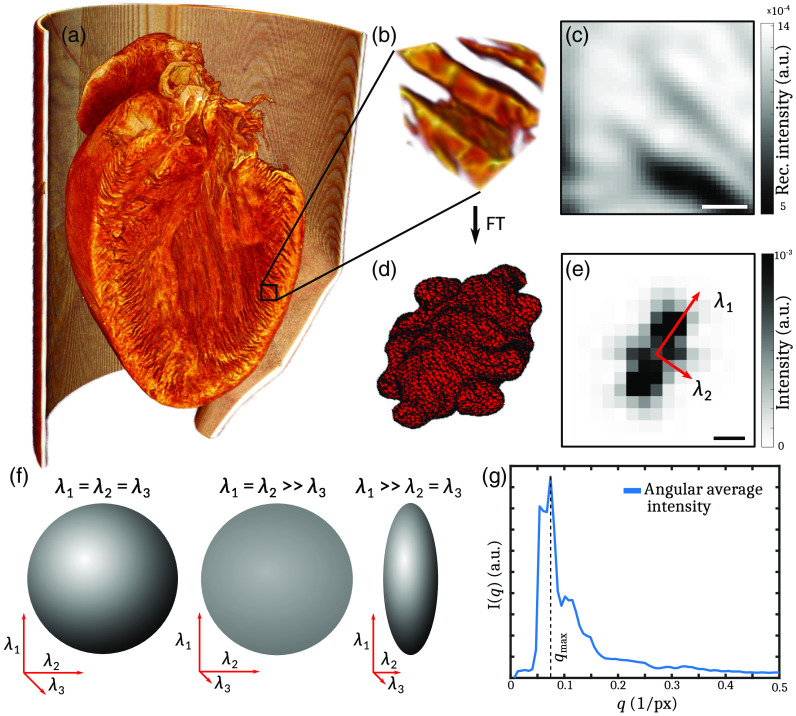
Workflow of the fiber tracking algorithm. (a) Volume rendering of the reconstructed electron density of a mouse heart treated with the EOS method. (b) Small cubic subvolume with a defined edge length. (c) 2-D slice of the subvolume. Scale bar: 50  μm. (d) 3-D Fourier space of this volume. By comparing the eigenvalues of the PCA, a degree of anisotropy in each subvolume can be defined. (e) 2-D slice of the Fourier transform. Sketch of two eigenvectors. Scale bar: $0.1\;\frac{1}{\text{pixel}}$. (f) Possible shapes of the ellipsoid spanned by the results from the PCA: sphere, disk, and rod. (g) Angular average of the intensity of the Fourier space. Peaks appear along the direction of the main component and correspond to main fiber thicknesses in the real-space volume.

For the analysis of the heart structure, an algorithm based on a local 3-D Fourier transform was implemented in MATLAB (The MathWorks, Inc.). It is based on the reconstructed electron density of the sample and delivers the fiber orientation, as well as the degree of fiber alignment and the local fiber thickness. Due to the clearly recognizable structure, the high SNR and resolution of the algorithm were applied on the heart which, was treated with the EOS method. Before the data were analyzed, the volume was filtered with a 3-D Gaussian function with a standard derivation of 1 pixel. The schematic workflow of the analysis is shown in Fig. 3.

The algorithm is applied to small subvolumes (probing volumes) of the tomographic reconstruction, which are then shifted through the entire heart. For each data point, a cube with a defined edge length l=48  pixel is virtually cut from the volume. Figure 3 shows (a) the volume rendering from the tomographic reconstruction carried out with Avizo (FEI Visualization Science Group), (b) a typical subvolume, and (c) a two-dimensional (2-D) slice of this subvolume. To reduce edge effects from the Fourier transform, the subvolume is multiplied with a 3-D Kaiser–Bessel window W(r) in the following form:

$$W(r)=\frac{I_{0}(\beta )\sqrt{1-{\left(\frac{r-l/2}{l/2}\right)}^{2}}}{I_{0}(\beta )},$$ (5)

with the zero-order modified Bessel function of the first kind $I_{0}(x)=\sum_{n=0}^{\infty }{\left[{\frac{x}{2}}^{n}/n!\right]}^{2}$, the distance r to the center of the subvolume, and the tuning parameter β=8. A typical outcome of the Fourier transform is shown Fig. 3(d). It is similar to a triaxial ellipsoid and yields information about the spatial frequencies in the volume. To quantify the orientation and degree of alignment of this ellipsoid, a principal component analysis (PCA) is applied to the covariance matrix C

$$C=(\begin{array}{ccc} \left\langle q_{xx}\right\rangle & \left\langle q_{xy}\right\rangle & \left\langle q_{xz}\right\rangle \\ \left\langle q_{yx}\right\rangle & \left\langle q_{yy}\right\rangle & \left\langle q_{yz}\right\rangle \\ \left\langle q_{zx}\right\rangle & \left\langle q_{zy}\right\rangle & \left\langle q_{zz}\right\rangle \end{array}),$$ (6)

with

$$\left\langle q_{ij}\right\rangle =\frac{\int I(q_{x},q_{y},q_{z})q_{i}q_{j}d\overset{\to }{q}}{\int I(q_{x},q_{y},q_{z})d\overset{\to }{q}}$$ (7)

for each subvolume of the reconstruction. The corresponding eigenvalue problem

$$C\cdot {\overset{\to }{b}}_{i}=\lambda_{i}\cdot {\overset{\to }{b}}_{i}$$ (8)

yields three eigenvectors ${\vec{b}}_{i}$ with i∈1,2,3, which form a new orthogonal basis. The eigenvalues $\lambda_{i}=\sigma_{i}^{2}$ correspond to the variance along the three principal directions. All eigenvectors are sorted by the corresponding eigenvalues in descending order. Two eigenvectors are sketched in the 2-D slice of the Fourier transform in Fig. 3(e).

#### 3.1.1 Fiber orientation

The vector related to the largest eigenvalue describes the direction with the highest variance in Fourier space, i.e., the direction of greatest structural changes. In the present case, this direction is orthogonal to the elongated muscle bundles. Thus, the orientation of the fibers in real space is directed perpendicular to the pattern orientation. It corresponds to the eigenvector with the lowest eigenvalue. While the orientation is given, the direction (±) remains undefined due to the point symmetry in reciprocal space. The angles obtained from this analysis in Cartesian coordinates are transformed to the coordinate system of the heart as described in Sec. 2.3.

#### 3.1.2 Degree of orientation

The results of the PCA span a triaxial ellipsoid in the eigenspace with an orthogonal basis of eigenvectors. The relation of the eigenvalues gives information about the degree of anisotropy ω, defined as^38^

$$\omega =\sqrt{\frac{{\left(\lambda_{1}-\lambda_{2}\right)}^{2}+{\left(\lambda_{2}-\lambda_{3}\right)}^{2}+{\left(\lambda_{3}-\lambda_{1}\right)}^{2}}{2(\lambda_{1}^{2}+\lambda_{2}^{2}+\lambda_{3}^{2})}},$$ (9)

$$\omega =\sqrt{\frac{1}{2}[3-\frac{1}{\text{trace}(R^{2})}]}$$ (10)

with $R=\frac{D}{\text{trace}(D)}$ and the normalized diagonalized covariance matrix D from the analysis. Depending on the relation of the eigenvalues $\lambda_{k}$, the shape of the ellipsoid can be approximated by a sphere ($\lambda_{1}\approx \lambda_{2}\approx \lambda_{3}$), a 3-D disk ($\lambda_{1}\approx \lambda_{2}\gg \lambda_{3}$), or a rod ($\lambda_{1}\gg \lambda_{2}\approx \lambda_{3}$). A sketch of these shapes is shown in Fig. 3(f). If all eigenvalues are the same length and therefore indistinguishable, ω=0, the ellipsoid degenerates to a perfect sphere. For ω=1, the ellipsoid becomes an infinitely long rod in the direction of the the largest eigvenvalue.

To maximize the anisotropy for a disk-shaped ellipsoid, which is the most relevant shape in Fourier space for fiber tracking, we modify the above definition according to $\lambda_{i}\to \lambda_{i}^{-1}$, defining a “reciprocal” anisotropy Ω

$$\Omega =\sqrt{\frac{{\left(\frac{1}{\lambda_{1}}-\frac{1}{\lambda_{2}}\right)}^{2}+{\left(\frac{1}{\lambda_{2}}-\frac{1}{\lambda_{3}}\right)}^{2}+{\left(\frac{1}{\lambda_{3}}-\frac{1}{\lambda_{1}}\right)}^{2}}{2({\frac{1}{\lambda_{1}}}^{2}+{\frac{1}{\lambda_{2}}}^{2}+{\frac{1}{\lambda_{3}}}^{2})}},$$ (11)

$$\Omega =\sqrt{\frac{1}{2}[3-\frac{1}{\text{trace}(R^{\prime 2})}]}$$ (12)

with $R^{\prime }=\frac{\frac{1}{D}}{\text{trace}(\frac{1}{D})}$. In this case, the anisotropy is 0 for the sphere shape, 1 for a disk-shaped ellipsoid, and 0.5 for an infinitely long rod. Hence, Ω is well suited to describe the strength of the orientation of the cardiomyocytes in the subvolume probed.

#### 3.1.3 Fiber distance

In addition, we stress that Fourier-based analysis in subvolumes processed in a sliding-window approach can go well beyond simple analysis of anisotropy since all structural information is contained in the phase and amplitude data. Figure 3(g) illustrates a simple example, showing a characteristic peak in the intensity distribution I(q) after a radial average. The peak at $q_{\max}=0.071\;\frac{1}{\text{pixel}}$ corresponds to a real-space periodicity of 14  pixel=74.2  μm, attributed to distances between adjacent cardiomyocyte chains. To highlight this signal, the signal was divided by a background curve computed from a cone-shaped integration along the direction of the shortest eigenvalue where no peak is observed.

### 3.2 Comparison with a Gradient-Based Algorithm

In the following, we compare the sliding-window Fourier method with the established gradient-based structure tensor (ST) analysis.^9^^,^^17^ The local ST is constructed from the first derivative of the intensity changes as

$$\mathrm{ST}=(\begin{array}{ccc} \sum g_{xx} & \sum g_{xy} & \sum g_{xz}\\ \sum g_{yx} & \sum g_{yy} & \sum g_{yz}\\ \sum g_{zx} & \sum g_{zy} & \sum g_{zz} \end{array}),$$ (13)

with $\sum g_{ij}$ sum of the products of the gradient along i and j within a cube with a certain edge length l around the central pixel. The analysis based on the gradient of intensities is similar to the Fourier analysis. The solution of the eigenvalue problem

$$\mathrm{ST}\cdot {\overset{\to }{b}}_{i}=\lambda_{i,\mathrm{grad}}\cdot {\overset{\to }{b}}_{i}$$ (14)

yields three eigenvectors ${\overset{\to }{b}}_{i}$ and eigenvalues $\lambda_{i,\mathrm{grad}}$, which also delivers information of structural orientation within the subvolume. The structures within the subvolumes are oriented toward the direction with the lowest changes in intensity. Thus, their orientation is also described by the eigenvector with the smallest eigenvalue. Furthermore, the reciprocal anisotropy of the ST is a measure for the alignment of the filaments within the volume as well.

**Fig. 4:**
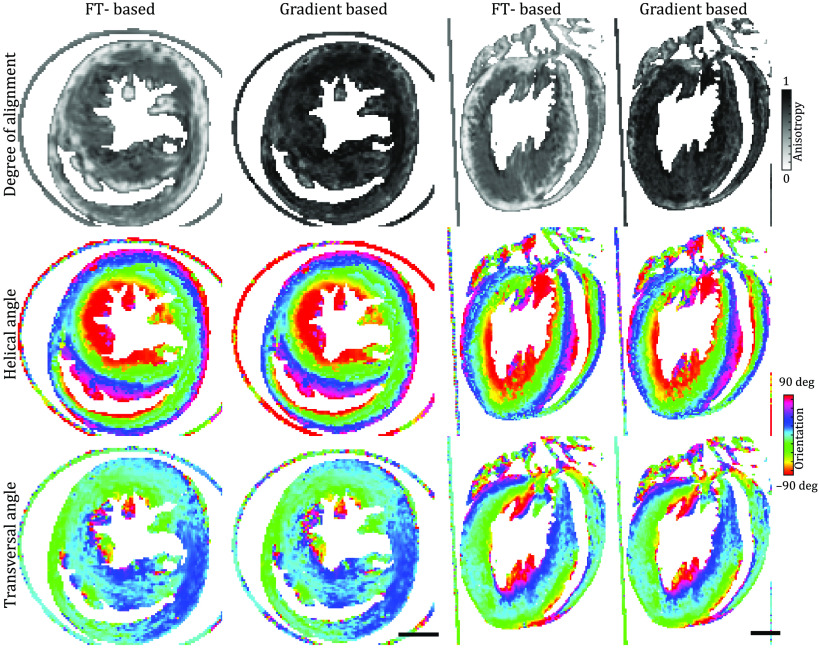
Results of the structural orientation obtained by the FT- and gradient-based algorithm. For each approach, the anisotropy, the helical angle, and the transversal angle of a slice along the long and short axes of the reconstruction are shown. Both algorithms provide similar results for the orientation of the inner structures of the heart. The values of the anisotropy, however, are systematically smaller for the Fourier-based approach. Scale bars: 1 mm.

The analysis of the structural orientation within the tissue was performed using the two different algorithms explained earlier. The results for the anisotropy, helical angle, and transversal angle are displayed in Fig. 4. Both algorithms show similar results. The values of the anisotropy, however, are systematically smaller for the Fourier-based approach, since the weight of Fourier components by (scattering) intensity is higher for smaller spatial frequencies. In fact, it is an advantage of the Fourier-based analysis that the window of q-values that are exploited for anisotropy analysis could be tuned by different weighting (or window) functions. A further concern is to match the real-space resolution or probing volumes of the two approaches. For the gradient-based approach, the gradient values are averaged over a cube of side length $l_{\mathrm{grad}}$. We match the real-space volume of the Fourier space approach, taking into account the Kaiser–Bessel window W(r), which is required to prevent aliasing effects, as

$$l_{\mathrm{grad}}^{3}=l_{\mathrm{FT}}^{3}\int drW(r).$$

In this work, a side length of $l_{\mathrm{grad}}=20\;\text{pixel}$ leading to $l_{\mathrm{FT}}=48\;\text{pixel}$ was chosen. The respective volumes were shifted by 10 pixels per analysis point. The application of the 3-D Kaiser–Bessel window leads to a smoothing of the edges and thus to small differences of the local information compared with the gradient-based approach. However, the extracted orientation from both algorithms is very similar. The results of a centered slice in the lateral and axial planes are shown in Fig. 4. Note that the gradient-based approach is computationally much faster since it is missing the calculation of the fiber distance.

By contrast, the FT-based analysis retains the spatial information of the structure in the subvolume. They are encoded in Fourier space and can be used to obtain information of the orientation of all structures within the volume as well as structural information within the subvolume, such as a dominant interfiber distance as described in Sec. 3.1. In the following, the results of such an analysis are presented.

### 3.3 Organization of the Three-Dimensional Heart Muscle Structure by Fourier Analysis

**Fig. 5:**
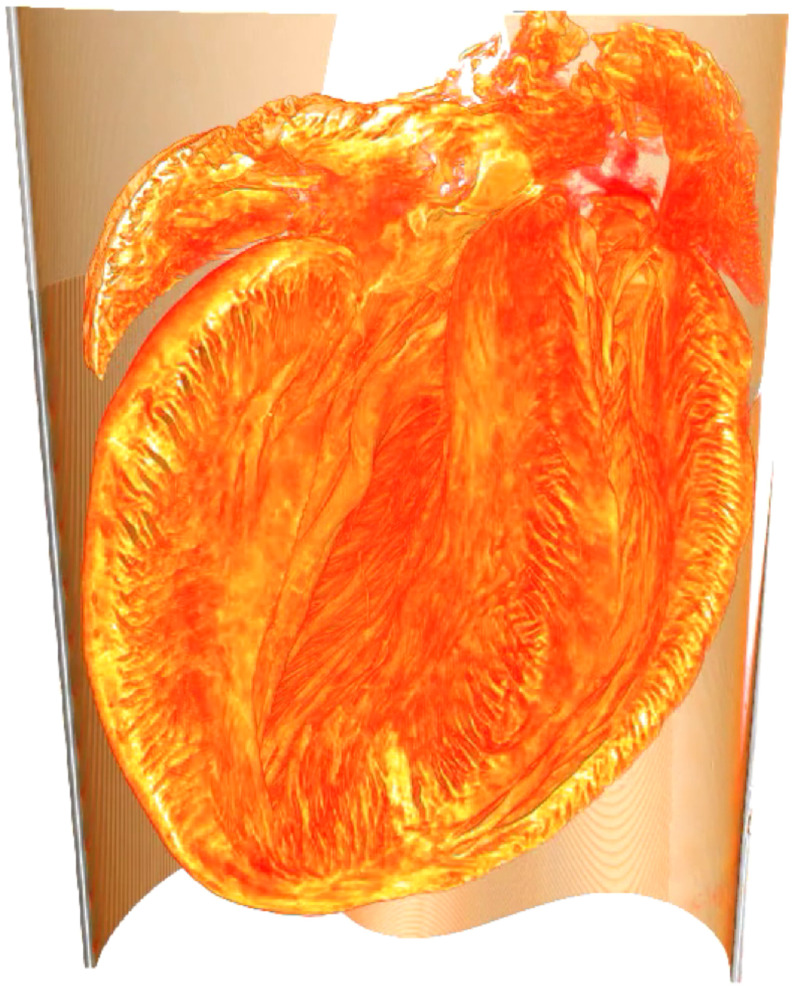
The volume rendering of the reconstruction of the electron density. Still image from Video 1 (Video 1, MP4, 10 MB[URL: https://doi.org/10.1117/1.JMI.7.2.023501.1]). For the video and the data files of the tomographic reconstruction and calculated vector field of the Fourier analysis, see also https://doi.org/10.5281/zenodo.3403379.

**Fig. 6:**
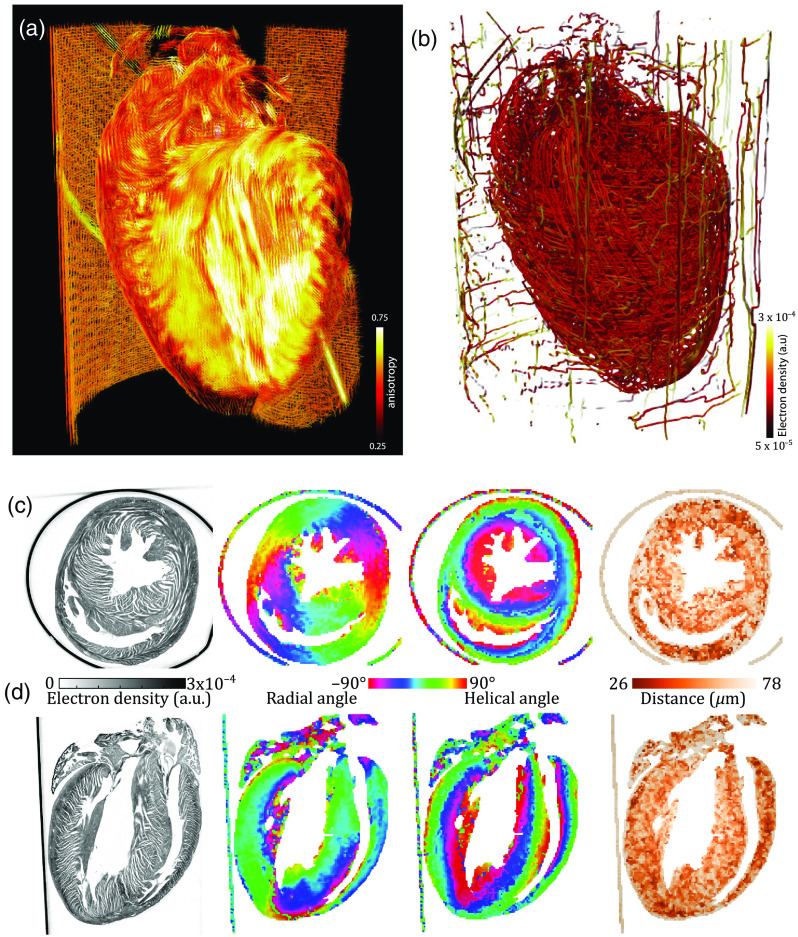
Results of the FT-based algorithm. (a) Virtual cut of the 3-D vector field from the FT analysis color-coded with the anisotropy. (b) Streamlines calculated from the vector field, (c) lateral and (d) transversal slice of the reconstructed electron density, radial angle, helical angle, and calculated fiber distance.

The reconstruction of the 3-D mouse heart structure obtained at the laboratory liquid metal setup, as shown in Fig. 5, was analyzed with the two different fiber tracking algorithms described earlier. The analysis of the heart was performed as sketched in Fig. 3 and the final results of the complete analysis, including the 3-D vector field of alignment, degree of orientation, helical and radial angle, and fiber thickness, are shown in Fig. 6. The 3-D representations are created by Paraview (Kitware, Inc.). The anisotropy, i.e., degree of orientation, is color-coded in the 3-D visualization of the vector field in Fig. 6(a). For an improved display, the vector field is cut in the same way as the reconstructed electron density in Fig. 3(a). Based on the direction of the structures and its anisotropy, a stream-tracing algorithm implemented in Paraview was performed. It results in a complex mesh of traces, which is shown in Fig. 6(b). The traces are winding around the ventricle in a helical manner, but there are also paths crossing with a high helical angle. To give a more ordered impression of the structure, two orthogonal slices of the heart are shown in Figs. 6(c) and 6(d). On the left, the reconstructed electron density of the 2-D slices is shown. The structure of the muscle tissue can clearly be derived from the background. Furthermore, the surrounding polyimide tube with a higher electron density can be identified. The middle left shows the radial angle obtained from the analysis. It indicates that the cardiomyocytes are arranged in a loop surrounding the ventricle. In the middle at the right, the slices are color-coded by the the helical angle. The value of the helical angle decreases from epicardium to endocardium. It is ∼20  deg and decreases to almost −90  deg near the left ventricle. Thus, there is a borderline in the middle of the myocardium around the left ventricle where the helical angle is close to zero. Consequently, the structures are oriented in the radial plane. The combination of radial and helical orientation indicates an arrangement of cardiomyocytes in a closed loop in the radial plane. This supports the old concept of the “Triebwerkzeug” by Krehl,^39^^,^^40^ according to which a strong muscle ring is surrounding the left ventricle to assist the contraction of the heart. In addition, the thickness of the structures was determined by the procedure explained above; see the color-coding in slices displayed on the right. It indicates a smaller interfiber distance in the apex of the heart and in the wall of the right ventricle. Note that the reconstructed distance highly depends on the preparation and dehydration of the tissue. Nevertheless, the thickness of the polyimide tube with a wall thickness of 75  μm can be exactly reconstructed by the algorithm.

### 3.4 Outlook

In this work, we have imaged whole mice hearts by propagation-based phase contrast using a laboratory μ-CT. The contrast of the reconstruction was found to be highly dependent on the preparation of the tissue. Metal-based stains enhance the contrast, but the specificity of the binding to the muscle tissue depends on the staining agent. In this work, PTA provided a more homogeneous and specific staining result than the iodine stain. Importantly, we have also shown that preparation without any staining agent, notably embedding in paraffin, ethanol, and especially the EOS method, resulted in satisfactory tomographic reconstruction of the heart muscle network. Based on these reconstructions, the orientation of muscle bundles was analyzed by two different algorithms. The gradient-based approach enables the possibility of determining the orientation of the muscle fibers in a small volume. Its functionality and certainty were already proven by comparisons with diffusion tensor MRI and histology.^8^^–^^10^ By extending the analysis of the algorithm by the degree of orientation, i.e., the anisotropy Ω, a further important structural parameter was included.

The Fourier-based approach implemented in this work, which also operates on small subvolumes of the tomographic reconstruction, delivers a similar output for the orientation of the structures. The Fourier transform of the subvolume is a representation of the entire structural information contained in real space. It allows for extracting a number of parameters beyond the anisotropy based on PCA. For instance, peaks in the 3-D Fourier intensity pattern correspond to periodicities in the electron density and can be used to deduce distances between myocyte chains. These peaks appear in the angular average of the background-corrected Fourier pattern. In addition to the identification of peaks in Fourier space, this type of analysis enables further possibilities for extracting information from the data. For instance, the variance of the distribution of fiber thicknesses can be extracted. It is also possible to look for specific distances and analyze the direction of multiple features by applying different q masks and performing multiple PCA. For example, it would be possible to analyze the sarcomere period as well as the thickness of the cardiomyocytes for high-resolution scans. Furthermore, it is possible to find multiple maxima in Fourier space and identify the orientation of multiple fibers in the subvolume, not only the average orientation for all structures within the volume.

Finally, it should be noted that the reconstruction with laboratory radiation at the present voxel size only allows for the extraction of the muscle fiber orientation in a rather indirect way since the different structural hierarchies are coupled. Small interfaces between aggregated units and/or connective tissue, and in particular small tissue walls opening up during the preparation, can serve as “reporters” of the underlying structural levels. Only by SR will it be possible to bridge whole heart imaging, for example, based on stitch tomography with the true capability to image individual myocyte chains. Further extension of this work will include application of the Fourier space algorithm to high-resolution SR, including the most suitable preparations in excess buffer, which avoids structural alteration by drying. The possibility of imaging the heart in buffer solution also opens the opportunity to analyze a beating heart using a Langendorff perfusion system.^41^ Future high-resolution 3-D imaging will be a fundamental requirement for the understanding of the contractile function. Building up on high-resolution data, local information about the structural parameters of heart tissue could be used to improve the understanding of heart function. For instance, results from the analysis could be used to model the electromechanical properties of the heart. Since the propagation speed of the neuronal signal is faster in the direction of fiber orientation, results from the presented analysis can improve existing computational models of the heart.^41^ By comparing the structure of healthy tissue with pathologies, such as in cardiac fibrosis, structural controls can also be developed for novel different diagnostic and treatment strategies.

## 4 Appendix: Online Supplemental Information

### 4.1 Video Showing the 3-D Reconstruction of Heart

For a video (.mp4) and the data files of the tomographic reconstruction and calculated vector filed of the Fourier analysis, see https://doi.org/10.5281/zenodo.3403379.
